# Modeling zebrafish escape swim reveals maximum neuromuscular power output and efficient body movement adaptation to increased water viscosity

**DOI:** 10.1016/j.isci.2025.112056

**Published:** 2025-02-17

**Authors:** Guillaume Ravel, Théo Mercé, Michel Bergmann, Anja Knoll-Gellida, Afaf Bouharguane, Sara Al Kassir, Angelo Iollo, Patrick J. Babin

**Affiliations:** 1 Department of Life and Health Sciences, INSERM, Maladies Rares: Génétique et Métabolisme (MRGM), U1211, University of Bordeaux, 33615 Pessac, France; 2 Team Memphis, INRIA Bordeaux Sud-Ouest, 33400 Talence, France; 3 University of Bordeaux, IMB, UMR 5251, 33400 Talence, France

**Keywords:** Kinematics, Neuroscience, Biophysics

## Abstract

Under evolutionary pressure, the kinematic and energetic characteristics of animal locomotion have been optimized for survival. We investigated the kinematics and energetic performance of zebrafish eleutheroembryo escape swims triggered by electrical stimuli in fluids of increasing viscosity. Eleutheroembryos exhibited a decrease in both tail movement frequency and swimming velocity in more viscous environments, while the amplitude of body curvature remains constant. We then combined experimental imaging of freely swimming eleutheroembryos with Navier-Stokes numerical simulations. The results showed that the mechanical power output was initially maximal and remained essentially stable with increasing viscosity, while the cost of transport was linearly correlated with viscosity. Eleutheroembryos maximize neuromuscular power output during the fast-start escape response, enabling them to potentially escape predators under all circumstances in a natural environment. This model may be used to identify genetic and toxicological factors that reduce the mechanical power developed by the neuromuscular system or induce a loss of efficiency in its use.

## Highlights

• During electric pulse-induced escape swims, the mechanical power output is maximal
• The same neuromuscular power output is delivered regardless of fluid viscosity
• The energetic cost of transport is linearly correlated to viscosity
• Factors inducing neuromuscular disorders may be investigated with the model

## Graphical abstract

**Figure:**
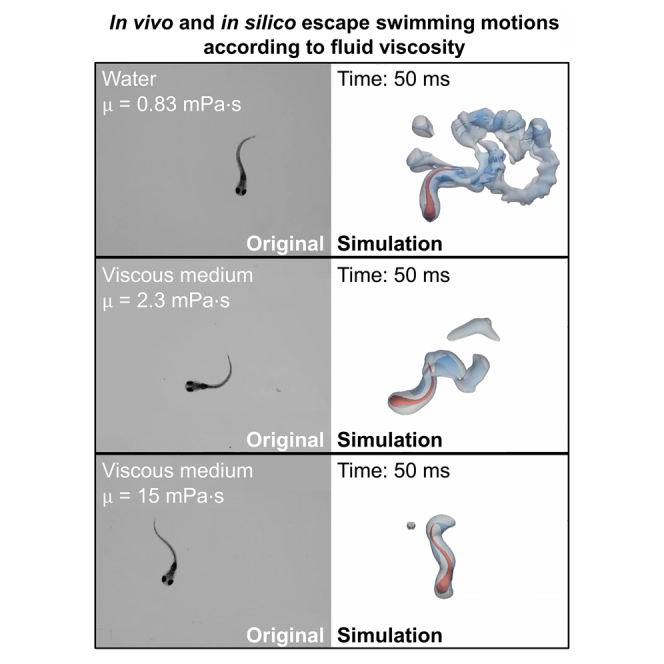

## Introduction

Animals can modify characteristics of their locomotor behavior according to the mechanical constraints exerted by the environment in order to optimize performance and increase survival. This physiological adaptability depends on the animal’s ability to adjust locomotion depending on environmental variations, for example, relative to the force and direction of currents in water or wind in the air.

Zebrafish (*Danio rerio*) are widely used as a model for drug screening, toxicology, and genetics, as it allows for lower infrastructure costs and simplicity of supply and handling when compared to other *in vivo* models. The three-to five-days post-fertilization (dpf) zebrafish, called eleutheroembryo or pre-feeding larva, is an important model organism for behavioral and locomotion studies as it is already able to swim. Among locomotion tests utilizing zebrafish, the well-known escape swims, also referred to as C-starts (the ‘C’ referring to the initial-phase C shape consistently attained by the fish body during escape), fast-starts or motor/defense response, have substantially contributed to investigations into the behavioral effects of genetic locomotion disorders^1^^,^^2^^,^^3^ and toxicant exposure.^4^^,^^5^^,^^6^^,^^7^^,^^8^^,^^9^ Fast-starts have been extensively studied and constitute well stereotyped escape behavior composed of three sequential movement forms: a C-bend, a counterbend, and fast swimming.^10^^,^^11^^,^^12^

In their natural environment, fish are required to produce the most effective locomotor behavior to escape predators while swimming in currents, i.e., while undergoing variable surrounding hydrodynamic forces. Fluid viscosity can be a relevant factor in evaluating fish escape-swim efficiency under variable hydrodynamic forces. Any displacement/rotation of the fish is the resultant of the action/reaction principle and, therefore, if the external viscous forces increase, the force deployed by fish to move in the medium is expected to increase. The impacts of fluid viscosity on adult zebrafish escape swims have been evaluated regarding kinematics, i.e., distance traveled, bending, and duration of the fast-start swim stages.^13^^,^^14^ A reproducible, reliable, and easy-to-use method for inducing an escape response of zebrafish eleutheroembryo as a model of energy expenditure, however, was previously lacking. This probably explains why this model of energy use had never been challenged by experimental variations in swimming medium viscosity. Theoretically, increasing water viscosity could lead to greater requirements in terms of the neuromuscular power, energy, and force exerted to enable a fast-start escape swim,^13^^,^^14^ regardless of any habituation process and/or genetic background. Based on this assumption, a locomotion test in media of heightened viscosity might uncover locomotor dysfunctions that are less perceptible in standard water. Theoretically, increasing media viscosity should provide a zebrafish equivalent to human tests of muscle power during physical effort.

In the literature, the primary experimental techniques used in zebrafish locomotion studies include tracking algorithms,^15^^,^^16^^,^^17^ midline digitization,^12^^,^^18^^,^^19^ kinematics measurements, or particle image velocimetry.^18^^,^^19^^,^^20^ Although these approaches provide valuable support for the analysis of motion and to infer forces in the case of particle image velocimetry,^21^^,^^22^ they could not provide a direct assessment of neuromuscular performance.^23^^,^^24^

As for swimming simulations, numerical methods have widely been applied to study swimming performance, and to optimize relevant body movement laws and swimmer geometry.^25^^,^^26^^,^^27^^,^^28^^,^^29^ Simulations have also been implemented in engineering and bio-mimicry fields,^30^ as well as in biology-inspired robotics.^31^^,^^32^^,^^33^^,^^34^ Several groups have reported empirical observations corresponding to the optimal range of Strouhal number,^33^^,^^34^^,^^35^^,^^36^^,^^37^ which can be used to describe oscillating flow mechanisms in fish propulsion. For C-starts, computational fluid dynamics studies have highlighted that zebrafish maximize distance traveled.^27^ Some authors have suggested that increased amplitude of body bends/beats could result in faster swims, although at increased energy expenditure^27^^,^^38^ and cost of transport (CoT).^38^ In earlier studies of fish larvae C-starts, the Reynolds number, which helps predict the associated fluid flow patterns, has been varied with no *in vivo* experimental support.^38^ A comprehensive understanding of fish swimming biomechanics across various developmental stages and locomotor behaviors requires an approach that integrates multiple components, including the neuromuscular system involved and fluid dynamics.^38^ Developing a numerical model that assesses energetic aspects based on experimentally recorded swims should promote understanding of how different parameters influence swimming performance and the associated energetic constraints. Previous reports have already demonstrated the interest of numerical simulations in quantifying the influence of drugs on spontaneous swims in zebrafish larvae.^39^^,^^40^

Fish movement feedback has been included in elastic or neuromechanical modeling,^41^^,^^42^ as well as in numerical deep reinforcement learning.^43^^,^^44^ Relatively few studies have included numerical solution of Navier-Stokes equations to enhance understanding of zebrafish swimming even though relevant information can be extracted from mathematical modeling.^38^^,^^45^ Despite considerable advances to close the gap between *in vivo* experiments and numerical simulations,^38^^,^^45^^,^^46^ using experimental data as input to a simulation of fast-swimming behavior has not been reported to date.

In the present work, we investigated the impact of increased fluid viscosity on 5 dpf zebrafish eleutheroembryo fast-escape responses in terms of kinematics and energetic performance. To this end, we implemented transdisciplinary approaches for simulation-based quantification of energetic performances from recorded zebrafish locomotion experiments. The simulation eleutheroembryo three-dimensional (3D) body shape was reconstructed from a database of transverse histological sections.^47^ Our experimental process relied on electric field pulses (EFP) to initiate stereotyped zebrafish escape responses. This emerging technology, using zebrafish eleutheroembryos, is the EFP motor response test (EFPMRT),^8^^,^^9^ which assesses the neuromuscular circuits of escape swims triggered independently from sensory functions.^48^ Unlike previous studies where fast-starts were engaged by using tactile^2^^,^^3^^,^^12^ or visual and acoustic stimuli,^1^^,^^11^^,^^13^^,^^14^^,^^49^ EFP stimulation allows experiments to be carried out with fish either individually or in groups using highly reproducible electrical stimuli that directly induce escape responses. This escape reflex is triggered by activation of the Mauthner cells (M-cells), without involving the sensory system.^48^ Recently, the EFPMRT has been employed to investigate the effects of various organophosphate molecules on zebrafish neuromuscular functions.^8^^,^^9^

To compute fish energetic performances, we have also developed an experiment-driven approach to reproduce filmed zebrafish escape responses *in silico* through the integration of Navier-Stokes fluid equations.^24^^,^^27^^,^^46^^,^^50^ In the present work, we applied our methodology to investigate fluid viscosity’s effect on 5 dpf eleutheroembryos EFP-induced escape response. We assessed performances of both observed and hypothetical body movements, respectively, while experimentally or virtually increasing fluid viscosity to challenge the escape response. We thus discovered information regarding the zebrafish’s ability to modify its escape behavior according to fluid viscosity, while maintaining maximum mechanical power that sustained optimal swimming performances.

## Results

### Numerical simulation of zebrafish C-start escape response

**Figure 1:**
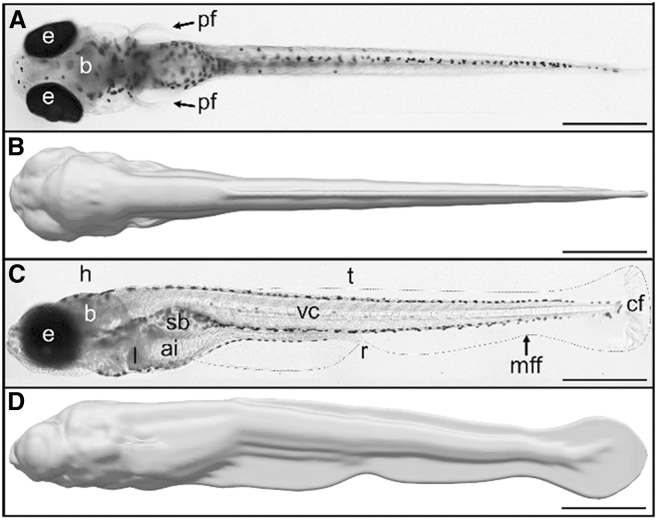
3D reconstruction of the zebrafish eleutheroembryo Comparison of 5 dpf zebrafish morphology under a combination of transmitted and incident illumination (A and C) to the *in silico* 3D reconstructions (B and D). The eleutheroembryo shape under the microscope and its digital counterpart are shown in dorsal (A and B) and lateral views (C and D). The median fin fold (mff) is highlighted by a dashed line in (C). Other abbreviations: ai, anterior intestine; b, brain; cf., caudal fin; e, eyes; h, head; l, liver; pf, pectoral fin; r, rectum; sb, swim bladder; t, trunk; vc, vertebral column. Scale bar: 500 μm.

**Figure 2:**
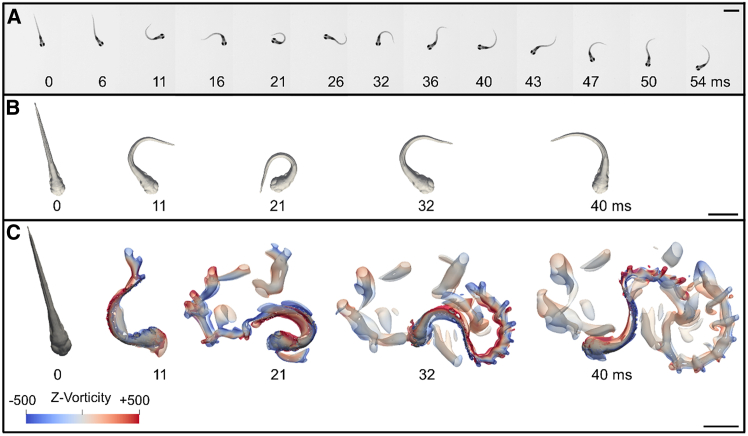
An experiment-driven numerical approach generated a digital reproduction of the zebrafish escape response triggered by an EFP stimulation (A) Snapshots of an experimental motor response of the first six tail bends/beats recorded at 10,000 frames per second. This escape motor response was a complex behavior composed of three sequential movement forms: a high-amplitude bending of the tail and body (C-bend) (0–11 ms) followed by a high-amplitude counterbend (11–21 ms) and a bout of fast swimming with regular amplitude beats (21–54 ms) to move away from the mimetic predator. (B) Body movements used as input to the Navier-Stokes solver. From swimming videos (A), image segmentation and Procrustes analysis isolated fish body movements from rotational and translational displacements. The virtual 3D zebrafish was then bent over time to fit with the observed isolated body movements. Resulting 3D snapshots were displayed for the maximal body curvature of the first four tail bend/beat cycles. (C) Flow generation during escape response numerical simulations. Fluid flow was visualized as the iso-contour of the Q-criterion colored by fluid vorticity in rotations per second (sˆ-1) along the vertical axis (z axis) at the indicated points in time. The complex escape motion (C) was computed from the actual input-based isolated 3D body movement snapshots shown in (B), reproducing the experimental dynamics (A). Aside from the strongest vortex in the fish wake, there were many smaller secondary vortex structures. They may have been caused by the lack of smoothness in the second derivative (acceleration term) of the recorded body movements. The smoothness of the image acquisition could indeed have influenced the simulation since the image acquisition time was about ten times longer than the timestep of the simulation. Panel B shows the fish body movement with translation and rotation subtracted by Procrustes analysis. As a consequence, the yaw angle (heading) was different from that of the real fish swimming in Panel A. Panel C corresponds to the Navier-Stokes solver, which recalculated the translation and rotation motions, explaining why the yaw angles were consistent with those of panel A, but not with those of panel B. On the other hand, the body curvature at each time point remained the same in all three panels. Scale bars: 1 mm.

In order to determine the swimming performances of zebrafish escape responses, we used filmed body movements as input for numerical simulations to obtain an experiment-driven digital equivalent. To this end, we first reconstructed a realistic 3D virtual 5 dpf zebrafish eleutheroembryo (Figure 1) based upon a transverse histological slice imaging database^47^ (see STAR Methods). Experimental imaging data (Figure 2A) of the first six fast-start swim tail movements of 5 dpf zebrafish during the EFP-induced escape response (first the C-bend, second a counterbend, followed by four fast swimming tail-beats) were processed through segmentation and Procrustes analysis. Procrustes analysis isolated body movements and nullified displacements (translations and rotations) (see STAR Methods). The resulting actual fish movements isolated from the associated translations and rotations were then used as a template to realistically bend and distort the virtual 3D zebrafish (Figure 2B). The resulting virtual 3D body movements were used as input for the Navier-Stokes solver (see STAR Methods). The numerical integration model computed both fluid and zebrafish motion, with quantification of mechanical power output and energy expended over time. In this manner, we obtained an *in-silico* reproduction of each stage of zebrafish escape response after an EFP stimulation. After two high-amplitude rotation and bending stages corresponding to the initial C-bend (*t* = 0*−*11 ms in the example of Figure 2A) and counterbend (*t* = 11*−*21 ms), zebrafish propelled themselves forward during several fast-swimming beats (*t* = 21 ms*−*end) analogous to acceleration of the mass center. These escape simulations also displayed the flow generated in the surrounding medium by the body movements (Figures 2C and Video S1). Some fluid was pushed away by the tail during escapes, or more precisely, by the very thin surface of the median fin-fold (Figure 1C). Video S2 illustrates the main steps of video processing: from the recording of a fish swim in water (top panel, left), to the extraction of body movements and *in silico* reconstruction of the 3D escaping fish model isolated from rotational and translational displacements (top panel, right, insert). Our data demonstrated that the wake generated during a C-start escape response in water (Figure 2C, Video S1, and Video S2 top panel, right) was consistent with the classical two rows of vortices generated by anguilliform swimmers in 3D.^.^ Finally, the Navier-Stokes simulation enabled computation of energetic parameters and displacements with fluid isovorticity structures shown alongside zebrafish (Video S1 and Video S2, top panel, right). Concordance between the observed swim kinematics (Figure 2A) and the virtual equivalent of the zebrafish escape response (Figure 2C and Video S2) was confirmed in terms of trajectory fitting and speed profiles (data not shown).

Video S1. Realistic digital equivalent of the zebrafish escape response after an EFP stimulation displaying the flow generated by body movements in the surrounding medium, related to Figure 2

**Figure 3:**
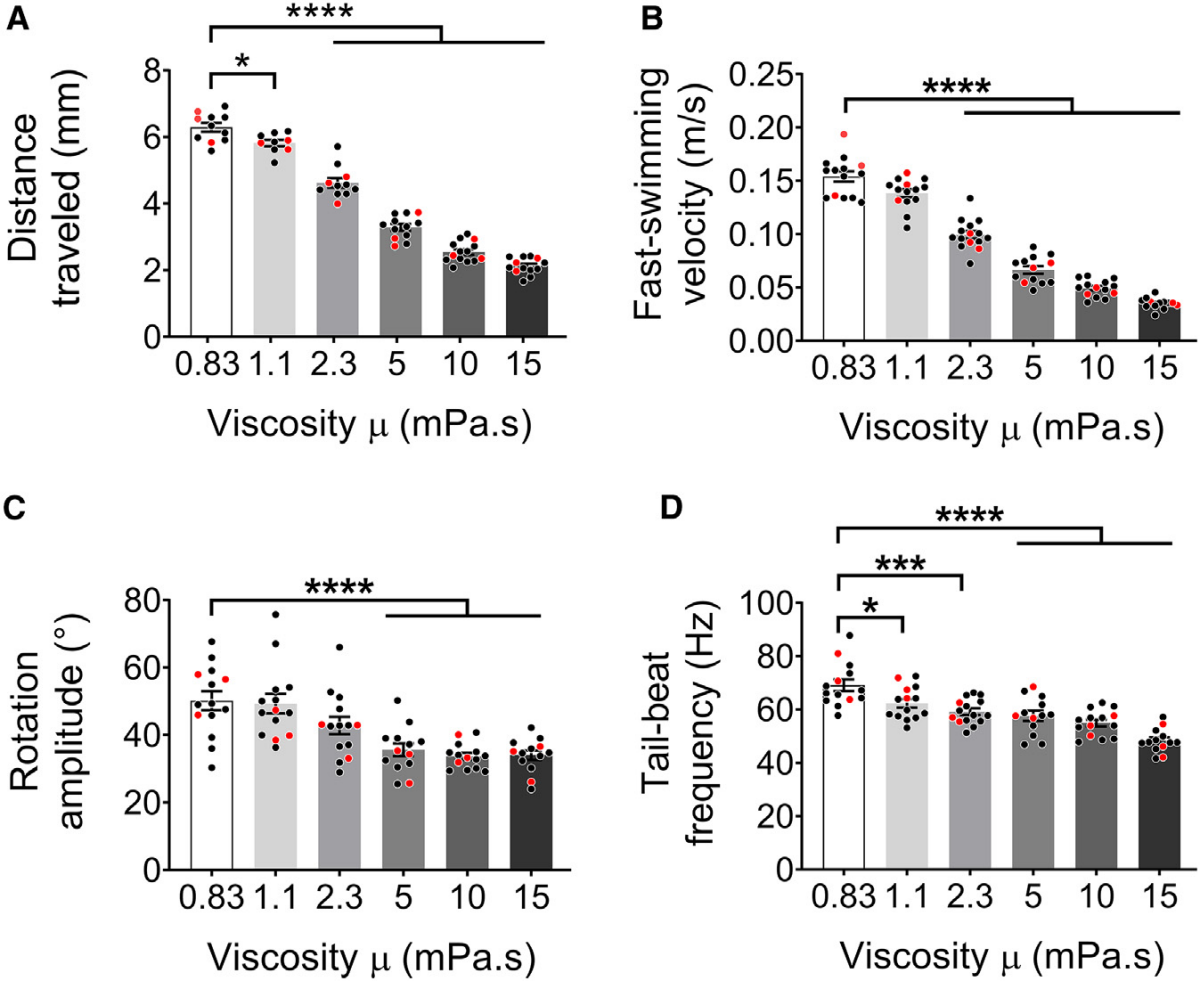
Swimming kinematics of zebrafish escape responses recorded in six viscosities between 0.83 (water) and 15 mPa⋅s (A) Distance traveled over six tail bend/beats. (B) Fast-swimming velocity during the fast-swimming stage, i.e., mean velocity during the two-fast-swimming cycles after the C-bend and counterbend stages. (C) Rotation amplitude, computed as the average of rotation maxima during the escape swim. (D) Tail-beat frequency during the fast-swimming stage. Data represented correspond to mean ± SEM of 10–15 escape responses per viscosity condition. For each viscosity experiment, the three red points represent the individuals selected for the subsequent numerical simulations. Significance of differences in measured parameters was assessed by ANOVA followed by post-hoc Dunnett test; ∗*p* < 0.05; ∗∗*p* < 0.01; ∗∗∗*p* < 0.001; ∗∗∗∗*p* < 0.0001.

**Figure 4:**
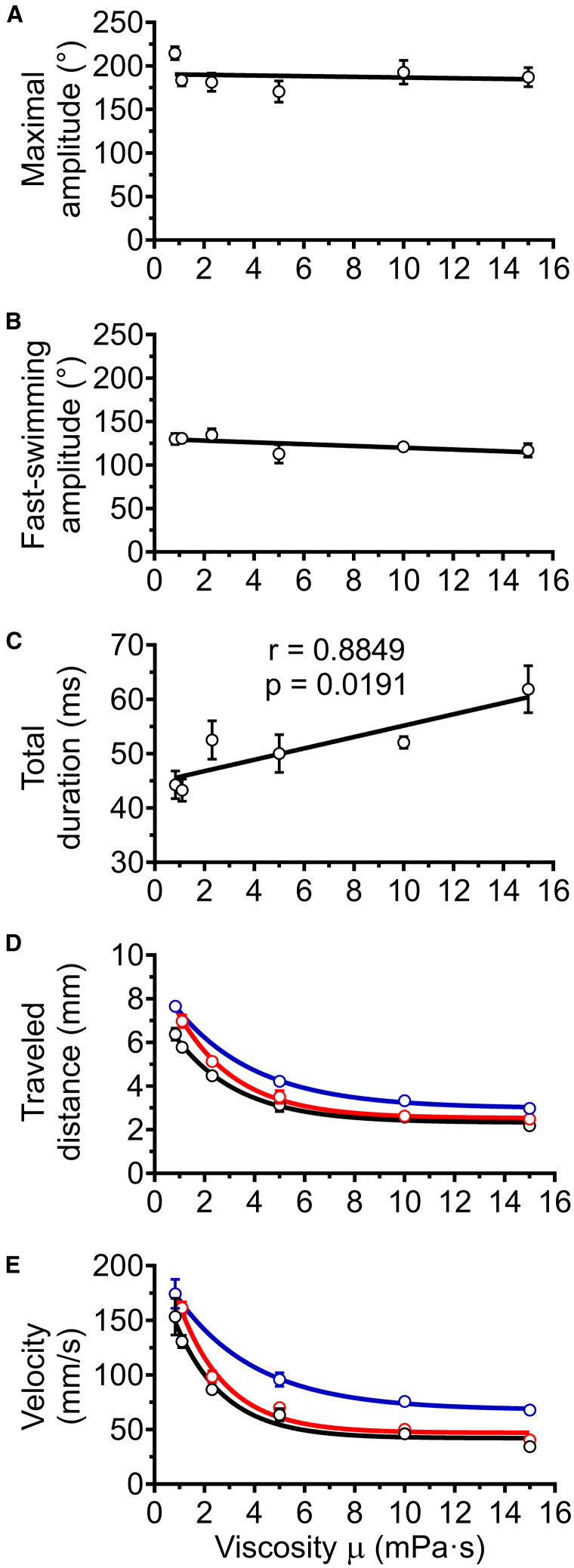
Kinematic analysis of zebrafish EFP-induced escape response across variations in experimental and *in silico* fluid viscosity Parameters were measured by kinematic analysis recorded from original movies (back lines) or through numerical simulations. Numerical simulations were performed with isolated body movements recorded in media of 0.83–15 mPa⋅s (red lines) or with the movements recorded in water (0.83 mPa⋅s) followed by computational enhancements of viscosity up to 15 mPa⋅s (blue lines). (A) Maximal head-tail angle, which measures the amplitude of the C-bend and counterbend, was not correlated with viscosity. (B) Fast-swim beat amplitude, defined as average amplitude of peaks during fast swimming, was not correlated with viscosity. (C) Total time for completion of the initial six tail bend/beats increased with viscosity. Total escape duration was significantly correlated with fluid viscosity as tested by Pearson’s test, and the linear regression slope was significantly different from zero (*p* < 0.001). Coefficient of determination R^2^ = 0.78 for the linear regression. For A, B, and C, the only line shown was extracted from kinematic analyses (black lines), because the midline bending values and the total duration required for six tail bends/beats were body movement descriptors that were used as input for the numerical simulations, and were therefore identical to them. (D) Distance traveled over six tail bend/beats measured through kinematic analysis on original videos and during simulations. Coefficients of determination R^2^ > 0.95 for both nonlinear regressions. (E) The average escape velocity, calculated over the duration of the six tail bends/beats, decreased exponentially with both experimental and *in silico* increases in viscosity. The coefficients of determination R^2^ > 0.90 for both nonlinear regressions. Data represented correspond to mean ± SEM of three escape responses per viscosity condition over six tail movements: C-bend, counterbend followed by four fast swimming tail-beats. The significance was tested using a two-way ANOVA and Sidak’s multiple comparisons post-hoc test. (D, E) The only significant differences between the measured parameters recorded from original movies (black lines) and movement simulations recorded in media of 0.83–15 mPa⋅s (red lines) occurred at the viscosities of 0.83 and 1 mPa⋅s (*p* < 0.01). In viscosities between 5 and 15 mPa⋅s, there were significant or close to significant differences (*p* < 0.039 to *p* < 0.057) in the value of these kinetic parameters between numerical simulations obtained from body movements recorded in media of different viscosities (red lines) and the simulations based upon movements recorded in water followed by virtual enhancement of viscosity up to 15 mPa⋅s (blue lines). See also Figure 3.

**Figure 5:**
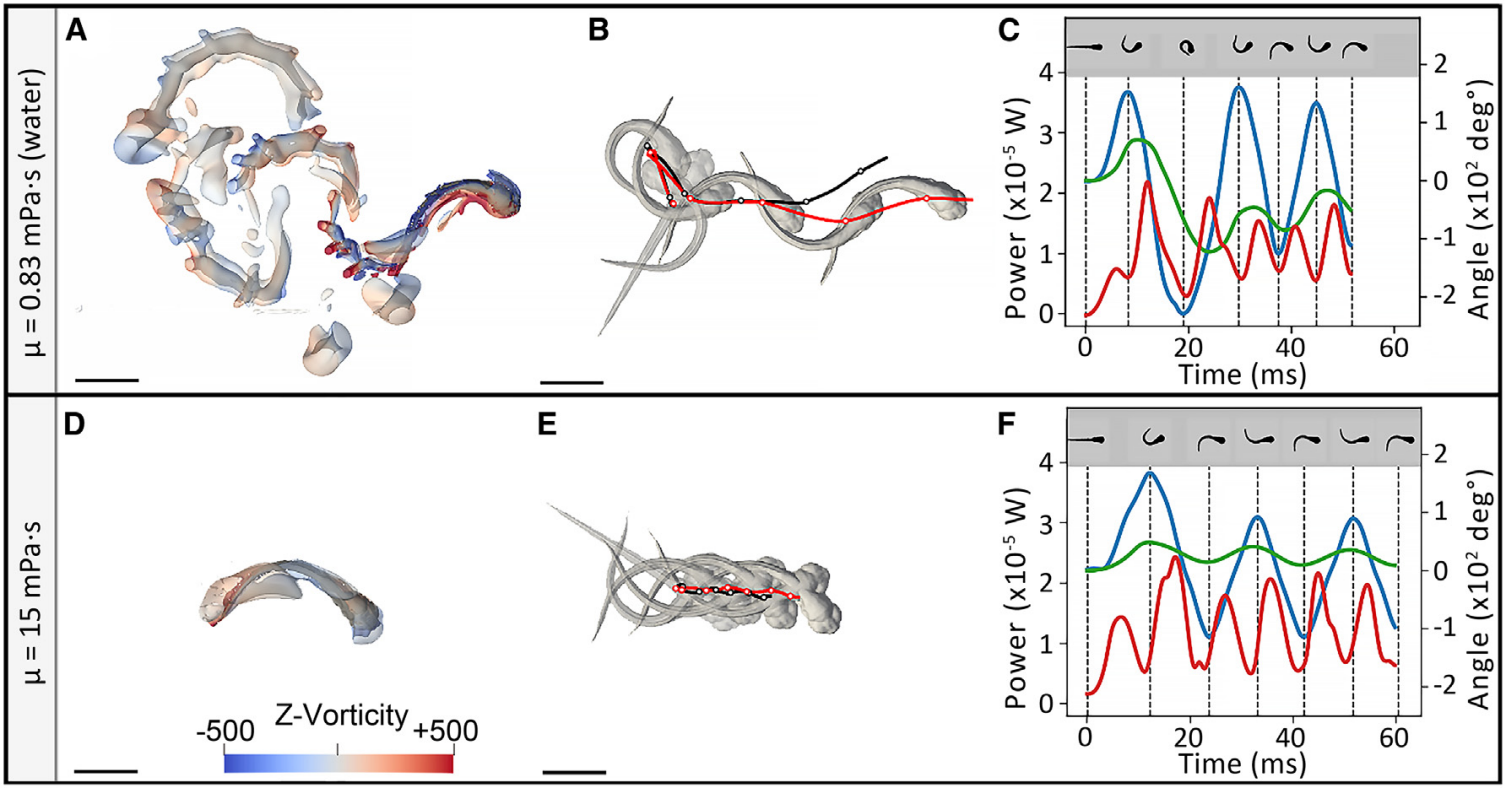
Swimming performance of zebrafish eleutheroembryo in water (μ = 0.83 mPa⋅s) (A–C) and in a high viscosity fluid (15 mPa⋅s) (D–F). (A and D) Fluid vorticity along the z axis after 1.5 swimming tail-beats (A) in water and (D) in a high viscosity fluid. Fluid flow was visualized as the isocontours of the Q-criterion colored by fluid vorticity in rotations per second (sˆ-1) along the vertical axis (z axis). (B and E) Corresponding trajectories obtained during simulation (in red) and kinematic analysis (in black). Zebrafish shapes at each body curvature maximum are shown in gray. (C and F) Associated power output (red curves) (x10^−5^ W) over time displayed along with body curvature, corresponding to the curvature of the midline (blue curves), and the body rotation (green curves), expressed in both cases as angle values (x10^2^ deg°). The local maxima of the body movements are indicated as vertical dashed lines and corresponding shapes are depicted above. Scale bar: 1 mm. See also Video S2.

Video S2. Visualization of variations in flow regime, wake, distance traveled and tail bend/beat frequency in water of viscosity 0.83 mPa⋅s and in fluids of viscosities 2.3 and 15 mPa⋅s during EFP-induced escape swimming motions, related to Figures 2 to 5, Video S1 The original videos (left panels) recorded in 0.83 (top panel), 2.3 (middle panel) and 15 mPa⋅s (bottom panel) were processed through segmentation and Procrustes analysis to obtain a 3D reconstruction of body movements in which translational and rotational displacements were subtracted (insert in right panels). The 3D shapes, composed of 300 × 180 Lagrangian markers, were then entered in the Navier-Stokes solver to obtain realistic numerical simulations (right panels) in which translational and rotational displacements were re-calculated. In the simulations, fluid isovorticity structures are shown (colored according to speed in m.s−1) alongside the virtual zebrafish (red)

### Impact of fluid viscosity on C-start escape response

The swimming performance of 5 dpf zebrafish eleutheroembryos during EFP-induced fast-starts was assessed in terms of kinematics and energetics across a range of fluid viscosity from 0.83 mPa⋅s (water at 28°C) to 15 mPa⋅s. For each viscosity value, we first recorded up to 15 escape responses, and analyzed the first six tail movements of the fast-start response (Figure 3). We extracted several significantly impacted descriptors of escape kinematics compared to control animals recorded in water. Significant decreases in distance traveled by the center of mass and in tail movement frequency were observed at a fluid viscosity as low as 1.1 mPa⋅s (Figures 3A and 3D). The escape velocity decreased significantly in viscosities above 2.3 mPa⋅s (Figure 3B). Coherently with the constrained translation-mediating movements (Figures 3A and 3B), rotational amplitude began to decrease significantly at 5 mPa⋅s (Figure 3C). Interestingly, the maximal midline curvature, occurring during either C-bends or counterbends, and the average bending amplitude during the fast-swimming cycles did not change significantly as viscosity increased (Figures 4A and 4B, Figure 5, and Video S2). Therefore, although viscosity constrained displacements (translations and rotations) and the dynamics of zebrafish body movements slowed as viscosity increased, similar bend/beat amplitudes persisted.

To test the effects of viscosity on energetic parameters, three escape responses from each experimental level of viscosity were selected for numerical simulation (red points in Figure 3). The criteria for selecting the three escape responses included a stereotyped escape pattern, experimental imaging quality, initial immobility, and a planar escape response with no roll or pitch. Across the investigated viscosity range, similar to the rapid decay in the Reynolds number (Figure S1), which describes the fluid flow regime, wake, and flow development was dramatically reduced as fluid viscosity increased, particularly in highly viscous regimes (e.g., Figures 5A and 5D). The computed Re for 5 dpf zebrafish during the escape swim ranged between 10 (for high viscosities) and 900 (for low viscosities) (Figure S1). The changes in both locomotion behavior and wake flow can be seen for viscosities of 0.83, 2.3, and 15 mPa⋅s in the numerical simulation shown in Video S2.

The total duration needed for fish to complete the initial six tail bend/beats, and therefore the total duration of the simulation increased with viscosity (Figure 4C). This longer duration was consistent with the viscosity-dependent lower tail movement frequency (Figure 3D). The simulations were performed using the reconstructed 3D shapes of zebrafish from which were subtracted translational and rotational displacements. Therefore, kinematic parameters were extracted from the simulations in order to validate the accuracy of the numerical model compared to the values measured in the corresponding movies (Figures 4D and 4E). Our data demonstrated good concordance between measured parameters, e.g., distance traveled and mean velocity, recorded from original movies (back lines) and the same parameters generated in numerical simulations obtained using movements recorded in media of 0.83–15 mPa⋅s (red lines). It should also be noted that we found no evidence of habituation or fatigue upon kinematic analysis following multiple EFP-stimulations of groups of fish across the tested viscosity range (Figure S2). This result ruled out the possibility that other sensory stimuli might have altered the locomotor escape response during the fish incubation period required for the EFP stimulation procedure.

The mechanical power deployed over time during the C-bend, counterbend, and four subsequent tail beats was quantified by resolving the Navier-Stokes equations of each phase (Figure 5). Remarkably, zebrafish consistently exhibited six power output (red curve) spikes located between each pair of midline curvature maxima, i.e., in the middle of each midline curvature phase (blue curves, with maxima as vertical dashed lines) both in fish breeding water and in high viscosity medium (Figures 5C and 5F). Conversely, each local power expenditure minimum was concomitant with a body curvature peak, i.e., mechanical power deployment was minimal when switching from muscle contraction on one side of the fish to the other side. This could be due to neuromechanical phase lags. When compared to body curvature, fish rotational phases (green curves) were shifted toward the right side of the time axis in low viscosity, underlining the fact that rotation was a consequence of the inertia initiated by body bending (Figure 5C). This shift decreased with increased viscosity since the constraints on fish displacements increase (results not shown); no notable shift was observed at 15 mPa⋅s (Figure 5F).

**Figure 6:**
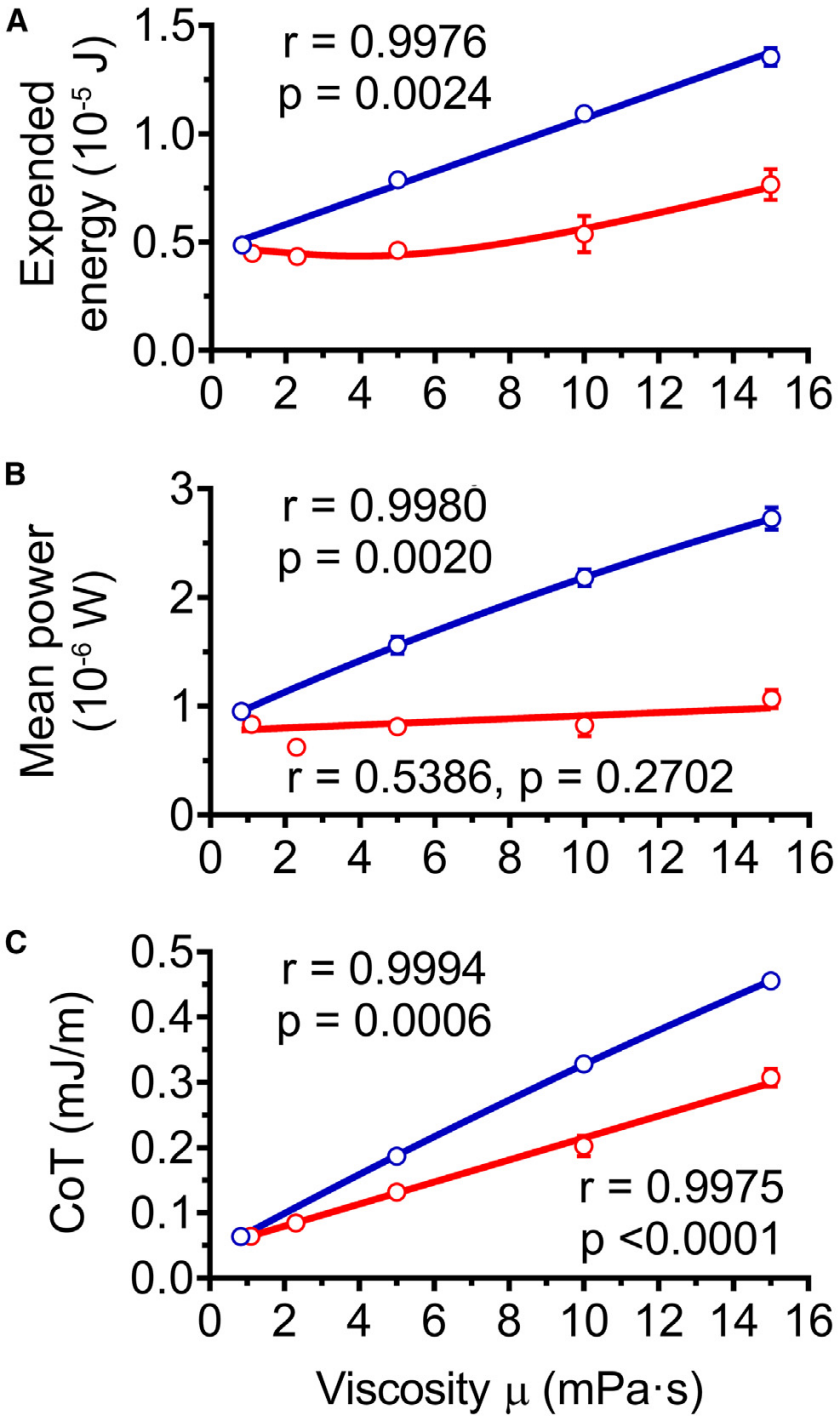
Energetic performances of zebrafish EFP-induced escape response according to increasing experimental and *in silico* fluid viscosities Energetic parameters were quantified after an experimental increase of viscosity using body movements recorded in media of 0.83–15 mPa⋅s (red lines). For comparison purposes, the same parameters were assessed after computational enhancements of viscosity up to 15 mPa⋅s by using body movements recorded in water (0.83 mPa⋅s) as input data (blue lines). (A) Total expended energy over six tail bend/beat cycles was not constrained. Experimental data-based simulations (red lines) showed no statistically significant change in total expended energy at viscosities lower than 10 mPa⋅s, and that the relation between expended energy and viscosity was not linear (*p* < 0.01 for 15 mPa⋅s). In the body movement simulations based upon recordings in water with virtual application of different viscosities (blue lines), a significant linear correlation existed with a regression slope significantly different from zero. Coefficients of determination R^2^ > 0.95 for virtual viscosity linear regressions and R^2^ = 0.7 for actual viscosity non-linear regression. (B) Mean power was not statistically significantly affected by actual experimental increases in viscosity. Experimental viscosity simulations (red line) showed no significant correlation between power and viscosity, and slope was not significantly different from zero. In contrast, the slope of the virtual viscosity simulations (blue line) was significantly different from zero and the corresponding Pearson correlation test was significant. Coefficient of determination was R^2^ > 0.99 for the virtual viscosity linear regression. (C) CoT was significantly affected by experimental differences in viscosity (*p* < 0.01 for 5 mPa⋅s, *p* < 0.0001 for 10 mPa⋅s and 15 mPa⋅s, respectively). CoT was linearly correlated with viscosity in experimental and virtual viscosity experiments. The slopes of both sets of simulations were significantly different from zero while Pearson’s tests showed significant correlations, but with a higher slope for *in silico* enhancement of viscosity (*p* < 0.0001). The coefficients of determination R^2^ were higher than 0.99 for both linear regressions. Data represented are mean ± SEM of three escape responses per experimental and virtual viscosity condition. Significance of differences in measured parameters was assessed by ANOVA followed by post-hoc Dunnett test.

Two descriptors were computed from the mechanical power output across the initial six tail movements, energy expenditure (Figure 6A) and mean mechanical power (Figure 6B). Strikingly, our *in-silico* experiments suggested that the mean mechanical power output was not significantly impacted by increased fluid viscosity (Figure 6B). On the other hand, the total energy expended during the first six tail movements was significantly higher at 15 mPa⋅s (*p* < 0.01, ANOVA followed by Dunnett test). Neither energy expenditure (Figure 6A) nor total time for completion of the first six movements (Figure 4C) was constrained given that both increased with viscosity. The CoT corresponds to the ratio of energy expended (Figure 6A) to the distance traveled (Figure 4B) and reflects the energetic cost required for escaping per unit length. As a mean value across the whole swim, the CoT also represents the mean power (Figure 6B) over the mean velocity (Figure 4D), i.e., the amount of neuromuscular power needed per speed unit, hence providing a relevant indicator of swimming efficiency. In terms of physical quantity, the CoT is also equivalent to the mean forces applied on the fish during its swim. Interestingly, CoT increased linearly with viscosity (Figure 6C). The linear relation between CoT and viscosity observed experimentally was preserved in the virtual viscosity experiments, although the slope of the latter was higher (*p* < 0.0001) (Figure 6C). This underlines the fact that with identical bend/beat amplitudes, the CoT was proportional to the viscous hydrodynamic forces experienced. Overall, these findings indicate that mechanical power is the primary biomechanical constraint during EFP-induced escape swims.

### Impact of computational versus experimental increase of viscosity on C-start escape response efficiency

For comparison purposes, we increased the viscosity of the medium in numerical simulations based upon movements recorded in water as input data (blue lines in Figures 4D, 4E, and 6). The power output, which was no longer biologically constrained, increased linearly throughout the virtual increase of viscosity (Figure 6B, blue line). Concomitant to this higher power expenditure, velocity rose with simulated viscosity increases compared to the experimental counterparts (Figure 4E). In addition, a slightly higher distance traveled was observed under simulated conditions (Figure 4D) and total energy expended over six tail bend/beat cycles was linearly correlated to *in silico* viscosity (Figure 6A, blue line). As could be expected from these elements, the CoT was significantly higher for each artificial viscosity simulation (Figure 6C, blue line) compared to simulations based on movements actually recorded in media of corresponding viscosities (Figure 6C, red line).

We hypothesized that compensating the diminution of tail movement frequency between experimental and computational viscosity simulations would eliminate the differences in mean power, speed and CoT. In terms of both power and energy, 5 dpf zebrafish escape responses recorded in water (0.83 mPa⋅s) and in a fluid of viscosity 1.1 mPa⋅s exhibited similar energetic expenditure as escape responses recorded in a 5 mPa⋅s fluid (Figures 6A and 6B). Before modifying tail bend/beat frequency, the isolated body movements recorded in 0.83 and 1.1 mPa⋅s were virtually immersed in a fluid viscosity of 5 mPa⋅s, constituting hypothetic simulations HS1 and HS2, respectively. A viscosity of 5 mPa⋅s corresponds to an intermediate flow regime in our experimentation (Figure S1). HS1 and HS2 were compared to realistic simulations (RS) of movements recorded in 5 mPa⋅s fluid. As shown in Figures 4 and 6 (blue lines), and 7C and 7F, HS1 was associated with significant increases in expended energy and mean power values, higher velocity, and a similar total distance traveled, resulting in a higher CoT when compared to RS (Figure 8, left panels). Similar quantitative variations in these parameters were observed for HS2 (Figure 8, left panels).

**Figure 7:**
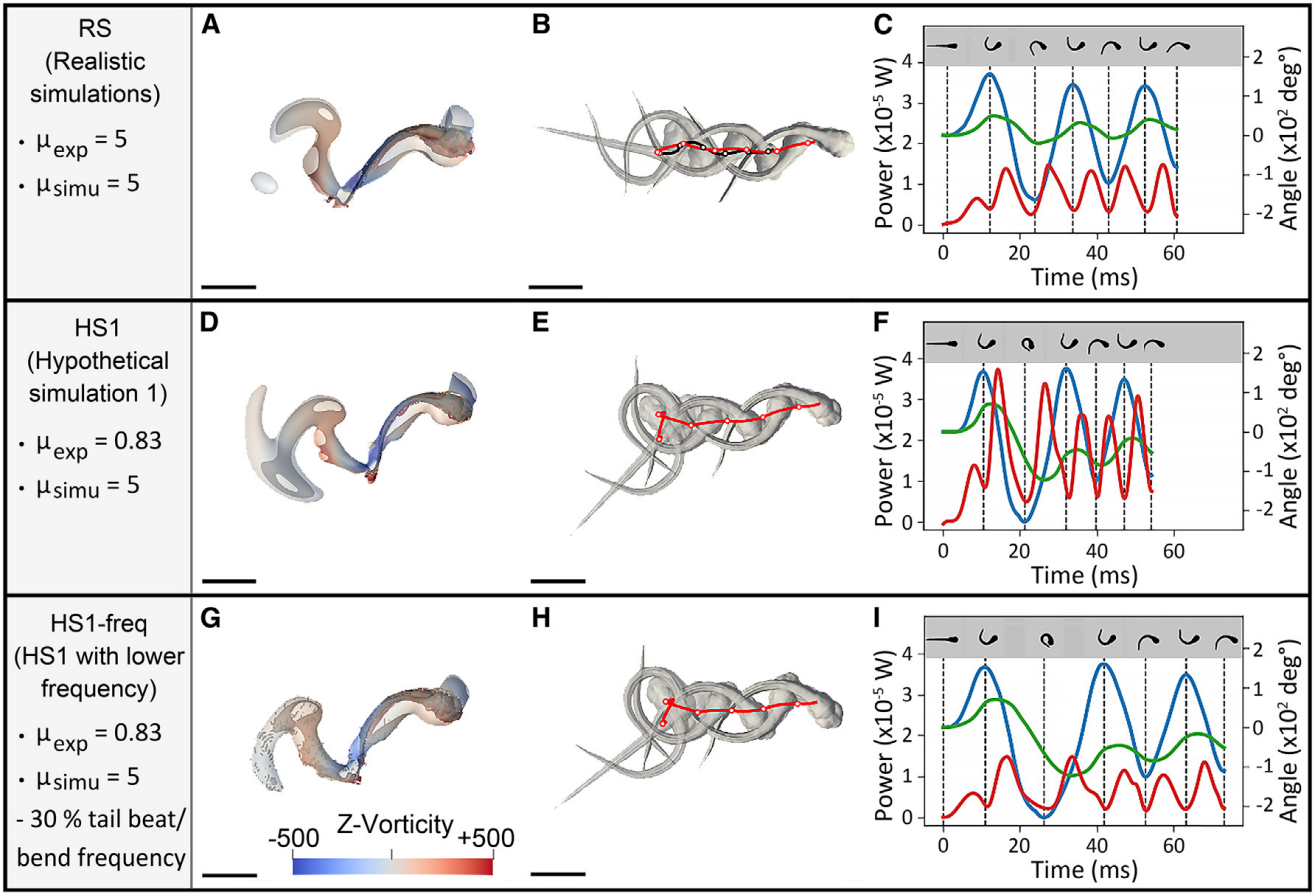
EFP-induced escape response swimming performances of isolated body movements recorded in media of 5 mPa⋅s as opposed to recordings in water, the viscosity of which was virtually increased to 5 mPa⋅s (A–I) Realistic numerical simulations (RS) of body movements were recorded in media of 5 mPa⋅s (A–C). Hypothetical simulations were obtained using movements recorded in water (0.83 mPa⋅s) followed by a virtual enhancement of viscosity to 5 mPa⋅s, before (hypothetical simulations 1 (HS1), D–F) or after (hypothetical simulations with lower frequency (HS1-freq), G–I) applying a 30% decrease in tail bend/beat frequency, which resulted in CoT, energy expenditure and distance traveled values that matched those of RS. (A, D, G) Fluid vorticity in rotations per second (sˆ-1) along the z axis after 1.5 swimming tail bends in realistic simulations (A) and in hypothetical simulations before (D) and after (G) slowing tail movement frequency. Fluid flow was visualized as the isocontours of the Q-criterion colored by fluid vorticity along the vertical axis (z axis). (B, E, H) Corresponding trajectories obtained during simulations (in red). Zebrafish shapes at each body curvature maximum are shown in gray. (C, F, I) Associated power output (red curves) (x10^−5^ W) over time displayed along the head-tail angle, i.e., body midline curvature (blue curves) and body rotation (green curves), expressed in both cases in angle values (x10^2^ deg°). The local maxima of isolated body movements are indicated as vertical dashed lines and corresponding shapes are depicted above. Scale bar: 1 mm. Experimental and simulated viscosities, respectively μ_exp_ and μ_simu_, in which fish motions were recorded or virtually computed, are specified as mPa⋅s (left panel). See also Video S3.

**Figure 8:**
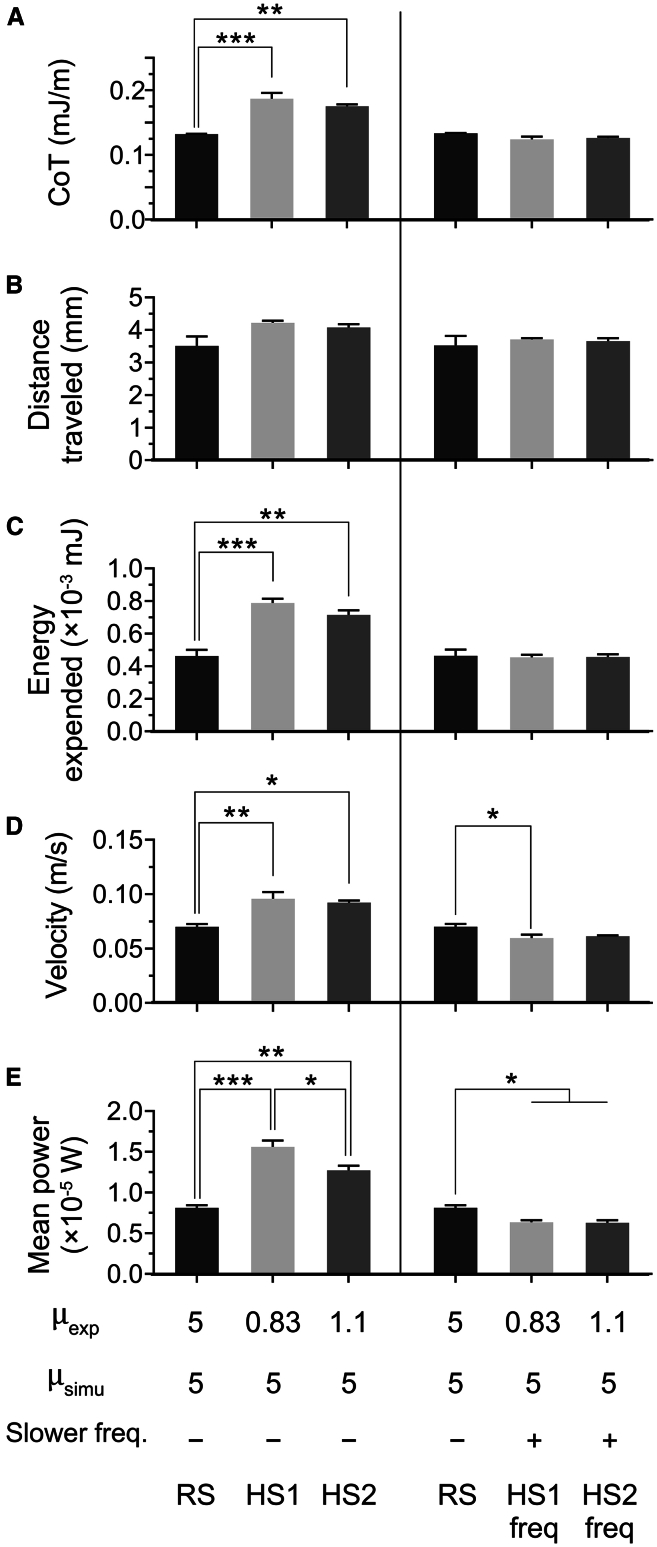
Energetic and kinematic analysis of EFP-induced escape response swimming performances of realistic simulations based upon recordings in μ = 5 mPa⋅s compared to simulations based upon recordings in μ = 0.83 or 1.1 mPa⋅s followed by virtual increases to 5 mPa⋅s (A–E) Realistic numerical simulations (RS) were obtained from movements recorded in media of 5 mPa⋅s viscosity. Hypothetical simulations were based upon movements recorded in viscosities of 0.83 mPa⋅s (HS1) or 1.1 mPa⋅s (HS2) as input, followed by a computational enhancement of viscosity to 5 mPa⋅s (left panels). Application of a hypothetical 30% decrease in tail bend/beat frequency (respectively HS1-freq, and HS2-freq) resulted in values that closely matched realistic CoT, energy expenditure and distance traveled (right panels). (A) In both HS1-freq and HS2-freq, the 30% frequency reduction brought CoT quite close to the corresponding RS value. Likewise, the hypothetical frequency reduction aligned all three values of distance traveled (B) and total expended energy over six tail bend/beat cycles (C). Swimming velocity (D) and mean power (E) were not aligned by the 30% frequency reduction. Data represented correspond to mean ± SEM of three realistic or hypothetical simulations. Experimental and simulated viscosities, respectively μ_exp_ and μ_simu_, in which fish motions were recorded or virtually computed, are specified as mPa⋅s (bottom panel). Significance of differences in measured parameters was assessed by ANOVA followed by post-hoc Tukey test; ∗*p* < 0.05; ∗∗*p* < 0.01; ∗∗∗*p* < 0.001. See also Figure 7.

Interestingly, a difference in mean power values between HS1 and HS2 was highlighted despite comparable CoT, distance traveled, energy expanditure and velocity (Figures 8A–8E, left panels), underlining the fact that mean mechanical power was not the sole determinant of escape velocity. Overall, the HS1 and HS2 escape-response swim velocities in virtual 5 mPa⋅s viscosity were faster than the realistic counterpart, i.e., RS, but at the expense of increased CoT and energy expenditure, originating at least partly from the corresponding unconstrained mechanical power that is exerted in the hypothetical simulations.

As the CoT corresponding to realistic simulations of body movements in a viscosity of 5 mPa⋅s was known (Figure 6C), we then applied a 30% diminution of frequency to HS1 (body movements in water simulated in fluid of 5 mPa⋅s) and HS2 (the movements in fluid of 1.1 mPa⋅s simulated in fluid of 5 mPa⋅s) and labeled those simulations HS1-freq and HS2-freq, respectively (Figure 8, right panels). Under the latter conditions, CoT, energy expenditure and distance traveled were similar to RS (Figures 8A–8C). Wake, trajectory and power findings associated with representative RS, HS1 and HS1-freq body movements appeared similar among RS (Figures 7A–7C), HS1-freq (Figures 7G and 7H) and HS2-freq (data not shown). An example of numerical simulations of RS, HS1, and HS1-freq and associated processing are displayed in Video S3. Interestingly, swimming velocity became significantly lower in HS1-freq compared to RS (*p* < 0.05, ANOVA followed by post-hoc Dunnett test), with a similar but nonsignificant trend for HS2-freq (*p* = 0.08) (Figure 8D) despite CoT matching (Figure 8A), which is likely a consequence of the reduced mean mechanical power of HS1-freq and HS2-freq (Figure 8E).

Video S3. In silico simulation experiments demonstrated an efficient body movement adaptation to high-viscosity fluids, related to Figure 6 to 8 Body movements were extracted through segmentation and Procrustes analysis from escape-swim videos recorded in μ = 0.83 mPa⋅s (original video not shown) or 5 mPa⋅s (original video in the top-left panel) and are displayed as a subpanel in the top right corner of each simulation panel. The hypothetical simulations (HS) in μ = 5 mPa⋅s were based upon body movements recorded in μ = 0.83 mPa⋅s before (middle panel, labeled HS1) and after (bottom panel, labeled HS1-freq) a 30% reduction in tail-beat frequency. HS1 had higher cost of transport, distance traveled, tail bend/beat frequency, mean speed, energy expenditure and power expenditure when compared to realistic simulations (RS) based on movements recorded in μ = 5 mPa⋅s. Decreasing tail bend/beat frequency by 30% (hence obtaining HS1-freq) aligned cost of transport, energy expenditure, and distance traveled values with those of RS. Strikingly, HS1-freq was associated with lower tail bend/beat frequency, speed and mean power expenditure, enlightening the fact that fish displayed temporal and spatial adaptations in body movements when viscosity was increased to μ = 5 mPa⋅s. Virtual enhancement of viscosity to μ = 5 mPa⋅s revealed body movement adaptations resulting in optimization of speed and cost of transport ratio during RS when compared to HS based upon body movements recorded in μ = 0.83 mPa⋅s. Fluid isovorticity structures are represented (colored according to speed in m.s−1) alongside virtual zebrafish (red)

## Discussion

Numerical simulations of fish swimming reported to date have mainly used experimental locomotion to validate the mathematical models.^38^^,^^39^^,^^42^^,^^45^^,^^46^ Biological experiments were the starting point for our approach because of the possibility of using the EFPMRT as a reliable and reproducible method for triggering locomotion. The zebrafish C-start escape response is a complex stereotypical escape behavior consisting of three sequential movements^10^ (Figure 2A). A fast, large-amplitude bending of the tail and body (C-bend) is followed by a large-amplitude counterbend and then by a bout of fast swimming with regular amplitude tail beats. EFP drives ultrarapid (order of magnitude, one millisecond) escape responses and induces well-coordinated swimming movements in zebrafish eleutheroembryos and larvae that strongly resemble C-start escape responses triggered by acoustic^10^ or mechanosensory stimuli^3^ (Figure 2, Video S2, and Video S4). Regarding C-starts, zebrafish kinematics, and previous estimations of forces, torques and power have been quantified using inverse-dynamics methods.^17^^,^^51^^,^^52^ Rather than considering real body movements, many advanced experimental-numerical computational fluid dynamics studies have focused on the heading angle.^38^ Alternatively, the computation of components such as translational and rotational velocity has been substituted by the experimental motion,^45^ which results in incompletely coupled fluid-structure interactions. In the present work, we computed rotational and translational displacements based on video imaging of actual body movements processed using Procrustes analysis. Thus, this experimental-numerical tool is well designed for animal experimentation involving locomotion as we are able to consistently reproduce realistic escape responses from multiple individuals, through simple modification of movement inputs.

Video S4. Kinematics of the touch-induced C-start escape response of a pre-feeding zebrafish larva, related to Figure S3 A tactile stimulation was performed on the upper aspect of the head to induce the fast-start escape swim. In the experimental system used, the animal moved through the water column in the X-Y plane with no visible pitch and roll that would have led to a significant deviation along the Z axis in relation to X-Y displacement. Video was acquired at a frame rate of 2,000 frames per second

### Mechanical power output during EFP-induced escape response is maximized and not significantly impacted as viscosity increases

Numerical modeling provides access to the energetic parameters of zebrafish eleutheroembryo escape responses induced by EFP and can thus highlight adaptations in terms of the kinetic parameters required according to fluid viscosity. Depending on the type of swim studied, e.g., spontaneous exploratory swim, visual motor response, escape/defense responses, the mechanical power measured does not necessarily correspond to the maximal neuromuscular power,^36^ which is of particular interest when studying locomotion disorders and movement coordination defects. It has long been hypothesized that zebrafish maximize their neuromuscular power output during escape behaviors due to the obvious evolutionary constraint to escape predators.^13^ This hypothesis is still debated given the short- and long-term habituation associated with acoustic or touch (mechano-sensory) stimulations.^53^^,^^54^^,^^55^^,^^56^^,^^57^^,^^58^^,^^59^ These studies have reported that, when used to induce escape responses, the mechano-sensory pathway is subject to central nervous system regulations that mediate the ability of healthy fish to learn from multiple consecutive stimuli.^53^^,^^54^^,^^55^^,^^56^^,^^57^^,^^58^ In contrast, EFP-induced escape responses are held to be independent from sensory regulation, because the EFP directly depolarizes M-cells.^1^^,^^48^ The initial escape and subsequent rapid swimming movements are initiated by M-cell activation that drives contralateral motoneurons in the spinal cord, as well as neurons in the nucleus of the medial longitudinal fascicle in the midbrain via activation of interconnected hindbrain cranial relay neurons.^58^ The ensuing swim away from the mimetic predator is driven by an alternating left-right rhythmic motor behavior activated by the spinal central pattern generator.^60^^,^^61^ Corroborating this hypothesis, we found no evidence of habituation or fatigue upon kinematic analysis following multiple EFP-stimulations of groups of fishes across the tested viscosity range (Figure S2).

This study showed how zebrafish eleutheroembryos respond to differences in fluid viscosity during an EFP-induced fast escape response. It could be hypothesized that fish need to deploy greater muscular power to escape when confronted with more viscous fluids. Numerical simulations carried out with body movements recorded in fluids from 0.83 to 15 mPa⋅s showed that the average mechanical power output was not significantly modified by increases in fluid viscosity (Figure 6B, red line). On the other hand, the total energy expended over the first six tail bends/beats was not constrained (Figure 6A, red line), as the time required to complete these swim cycles increased (Figure 4B). Increased fluid viscosity resulted in quantitative decreases in tail bend/beat frequency, translation, and rotation (Figure 3), while CoT rose (Figure 6C) and bending amplitude of body movements remained unchanged (Figures 6A and 6B). Together, these results highlight the fact that during EFP-induced fast-starts, the amount of biomechanical force deployed by the neuromuscular system of 5 dpf eleutheroembryos was maximized. This maximization of force might be achieved by activation of all the neuromuscular junctions in an optimized sequence. Furthermore, there was no limitation on the metabolic capacity of the neuromuscular system to assume the C-start response during the time window used for the experiments.

### The decreased tail bend/beat frequency does not fully account for the optimization of the C-start escape swim across increasing viscosity

The observed discrepancies in swimming energetic performance between experimental and *in silico* fluid-viscosity increases (Figure 6) could be explained by temporal or spatial differences in observed zebrafish body movements in the context of neuromuscular power constraints. Overall, the present realistic energetic simulations revealed that zebrafish exhibited changes in body movements in viscous media enabling escape swims at optimized CoT under power constraints. This, in turn demonstrated that fish escape swims were adapted to different flow regimes and different hydrodynamic forces.

Computational tools can support and refine biological experiments, generating insights and testing supplementary hypotheses about swimming mechanisms. In contrast to EFP-induced escapes, during which fish exert maximum mechanical power, spontaneous exploratory swimming does not require maximal use of available muscular power. In the latter situation, fish achieve higher speeds by increasing tail-beat frequency rather than amplitude, thereby minimizing the CoT.^24^ Application of methods used in the current study could help further understanding of how power production is shared spatially and across different phases of eleutheroembryo spontaneous exploratory swimming, the object of other recent simulation studies.^39^^,^^40^

Our analyses of fast-start escape responses indicate that tail bend/beat frequency does not entirely account for the differences observed between experimental and simulated viscosity changes (Figures 6, 7, and 8). Neither the maximal head-tail angle during the C-bend and counterbend movement forms, nor the average amplitude of fast-swim tail beats was correlated to viscosity (Figures 4A and 4B, and Video S2). The animal maintained a reflex escape C-bend movement of complete body reversal to allow an escape response in the direction opposite to that of the mimetic predator independently from external constraints.

Our experiment-driven computational approach allowed the assessment of mechanical power output related to real escape responses through simulated alterations in viscosity and body movements. We showed that during C-start escape swims, power outputs remained constant regardless of viscosity and consisted of power peaks correlated with body movements (Figures 5C and 5F). The fish maintained a constant mechanical power despite increases in the viscosity of the medium. Since power is the product of muscular force and the resulting speed, and power remained constant while speed decreased as viscosity increased, the exerted muscular force consequently rose proportionally with viscosity. The fish compensated for the increased fluid resistance by exerting greater force with each tail beat. To achieve this under its muscular power constraints, it reduced the number of beats per unit of time, effectively increasing the time needed to complete the six-tail bends/beats.

The constant amplitude of the tail-beats during the fast-swimming stage suggested that the fish did not alter the way they swam, but rather adapted the temporal dynamics of their movements to the viscous environment. Nevertheless, the numerical simulations suggested that the change in tail beat frequency was not the only factor involved in the production of additional mechanical force in a viscous environment (Figures 6, 7, and 8). Other processes that are also independent of the beat amplitude may have been involved. Fish can modulate whole-body flexibility by varying the timing and intensity of muscle activity on opposite sides of the body.^62^ However, little is yet known about muscle-mediated modulation of fish body stiffness during swimming or how sensorimotor feedback might be involved.

EFP stimulation leads to escape behavior initiated by M-cell activation. The induced rapid swimming sequence enabled fish to flee using the maximum mechanical power output that the fish can deliver for the duration of the escape swim sequence, independently of surrounding hydrodynamic forces. This highlights a likely evolutionary constraint represented by the need of fish to be able to perform optimized escape swims independently of either current strength or water temperature, which influences the viscosity. This also demonstrates the resilience of the central pattern generator, which induces contralateral activation-inhibition mechanisms during forward swims.^61^^,^^63^ In our tests, the central pattern generator mediated lower tail-beat frequencies to swim forward, optimally exploiting the available neuromuscular mechanical power according to external constraints.

### Potential identification of genetic or toxicological factors affecting mechanical power

Animal models such as zebrafish are particularly important in studies aiming to understand potential deleterious effects of genetic factors or chemical compounds on the nervous and/or neuromuscular systems involved in locomotor behavior. New approaches methods using this model can be used for human health risk assessment covering developmental and adult neurotoxicity, as well as for drug discovery.^64^^,^^65^^,^^66^^,^^67^^,^^68^ Our data supported the uncoupling of EFP-induced escape behavior from sensory escape-triggering functions that constitute confounding factors when assessing neuromuscular performance and complexify interpretations in other behavior assays used to evaluate disruptors specifically targeting the neuromuscular system. EFPMRT has already been used to assess the effects of organophosphate neurotoxicants on kinetic parameters of the zebrafish C-start escape response and to evaluate antidotes for cholinergic syndrome.^8^^,^^9^

The maximal power generation during fish escape movement is governed not only by the biomechanical properties and biochemical features of muscle cells, but also by the nervous system’s ability to appropriately activate muscles. The numerical model developed in the present study could be used to identify factors of genetic or toxicological origin likely to reduce the capacity of the neuromuscular system to produce or use maximum mechanical power. This would involve, for example, a reduction in the intrinsic neuromuscular and metabolic capabilities to produce this power, e.g., reduced muscular power due to altered morphological factors, such as muscle fiber type ratio, or reduction of metabolic capacity. Disruptions in either body flexibility or the coordination of trunk and tail undulations resulting in decreased mechanical power output or increased CoT might also be demonstrated.

### Conclusion

The present study demonstrated the previously hypothetical maximization of neuromuscular power output during the fast-start escape response of a teleost fish. The numerical simulations of this C-start escape response in high-viscosity fluids revealed that zebrafish locomotion delivered the same neuromuscular power output regardless of fluid viscosity. Remarkably, the present work also showed that the zebrafish eleutheroembryo was able to adapt its body movements in high-viscosity fluids enabling escape swims at optimized CoT under power constraints. Given that the production of maximum neuromuscular power after EFP stimulation is demonstrable with the zebrafish model, it can theoretically be used to characterize any factor disrupting neuromuscular performance, whether the disruption is related to reduced mechanical power output or a loss of efficiency in its use.

### Limitations of the study

The method developed is to be used with a planar escape response and limited rolling or pitching motions, in addition to the yawing motions of the zebrafish eleutheroembryo. In addition, the numerical simulations in this study incorporate actual body motions from experimental imaging to model escape responses for different fluid viscosities. However, they remain subject to certain limitations. In particular, the elastic responses of the body and musculature are not taken into account, potentially limiting the accuracy of force transmission and energy storage dynamics during rapid movements. In addition, potential sensorimotor feedback effects are not fully taken into account. However, these limitations do not appear to significantly affect the correspondence between numerical and experimental results in validation experiments.

## Resource availability

### Lead contact

Requests for further information on resources and reagents should be directed to and will be supplied by the lead contact, Patrick J. Babin (patrick.babin@u-bordeaux.fr).

### Materials availability

No new unique reagents were generated for this study.

### Data and code availability

• All data (and codes) reported in this paper will be shared by the lead contact upon request.
• Codes used in the current study can be found here: https://github.com/TheoMrc/zebrafish-procrustes-analysis; https://github.com/TheoMrc/zebrafish-3d-reconstruction.
• If you have any questions about the Navier-Stokes solver, please contact the author in charge of this aspect, Dr. Michel Bergman, at michel.bergmann@inria.fr.

## Acknowledgments

We thank Cédric Babin for his help during the first stages of the implementation of this project. Guillaume Ravel was supported by a predoctoral fellowship from the Projet IdEx Bordeaux/CNRS Projets Exploratoires Premier Soutien (PEPS). Théo Mercé was supported by a predoctoral fellowship from the French Ministry of Research and Education. The study was also funded by the Agence Innovation Défense (AID/DGA) via the Agence Nationale de la Recherche (ANR)-Accompagnement Spécifique des Travaux de Recherches d'Intérêt Défense (ASTRID), project N°ANR-21-ASTR-0023-01. This work was also carried out within the framework of the European Partnership for the Assessment of Risks from Chemicals (PARC) and has received funding from the European Union’s Horizon Europe research and innovation program under Grant Agreement No 101057014. Many thanks to the NeuroData group and David Grant Colburn Hildebrand who provided access to the high-resolution massive database located at https://neurodata.io/data/hildebrand16, based upon which we reconstructed zebrafish 3D morphology.^47^ All the numerical simulations were carried out using two computer french clusters: the PlaFRIM experimental parallel testbed, supported by 10.13039/100012950INRIA, LABRI, IMB, 10.13039/501100006251University of Bordeaux, 10.13039/501100024782Bordeaux INP, Conseil Régional d’Aquitaine, 10.13039/501100004794CNRS and the MCIA experimental testbed, CURTA, supported by the Pôle de Recherche et Enseignement Supérieur (PRES) of the University of Bordeaux and the 10.13039/501100022108Universities of Poitiers and the Pays de l’Adour Region (UPPA). We thank Jim Sneed for his careful review of the manuscript.

## Author contributions

P.J.B. and A.I. conceived the project. All authors designed the experimental and *in silico* procedures. G.R. and T.M. performed and optimized the numerical simulations. G.R. and A.K.-G. performed the *in vivo* experiments. G.R., T.M., A.K.-G., M.B., A.B., S.A.K., and P.J.B. analyzed the data. G.R., T.M., and P.J.B. wrote the paper with contributions from A.K.-G. All authors commented on the manuscript. Funding acquisition, P.J.B. and M.B.

## Declaration of interests

The authors declare no competing interests.

## STAR★Methods

### Key resources table

**Table:** 

REAGENT or RESOURCE	SOURCE	IDENTIFIER
**Chemicals, peptides, and recombinant proteins**		

Instant Ocean Sea Salt	Aquarium systems	Cat# 05120810
Calcium sulfate dihydrate, 99%	Alfa Aesar	Cat# 033301A1
Dextran 500	Pharmacosmos	Cat# 551005008006
Paraformaldehyde	Sigma Aldrich	Cat# P6148

**Deposited data**		

Raw kinematic and energetic data	This paper	Supplementary data
Videos	This paper	Supplementary data

**Experimental models: Organisms/strains**		

*Danio rerio*: WT (UB strain)	University of Bordeaux	N/A

**Software and algorithms**		

MATLAB	The MathWorks	https://www.mathworks.com/products/matlab.html
Python version 3.10	Python Software Foundation	https://www.python.org/
Scipy version 1.14	Python Software Foundation	https://scipy.org/
Procrustes analysis algorithm	This paper	https://github.com/TheoMrc/zebrafish-procrustes-analysis
3D reconstruction	This paper	https://github.com/TheoMrc/zebrafish-3d-reconstruction

### Experimental model and study participant details

#### Zebrafish husbandry

Wild-type UB strain zebrafish (*Danio rerio*) were raised inside our housing facilities in accordance with the French Directive (Ministère de l’Agriculture et de l’Alimentation) under permit number A33-522-6. Eleutheroembryos were utilized in the locomotion experiments at 5 dpf, at which point they are hatched but not yet free-feeding. This developmental stage was obtained by natural mating and raised in embryo water containing 86 μg/mL Instant Ocean (Aquarium Systems, Sarrebourg, France), 0.55 mM CaSO4, 2H2O (Alfa Aesar, CAS n°10101-41-4), dissolved in reverse-osmosis purified water, at 28 ± 1°C, with an 11 h light - 13 h dark photoperiod.

### Method details

#### Experimental setup

Zebrafish eleutheroembryos were positioned individually in the stimulation chamber 5 cm in diameter containing 4 mL of filtered fish water or viscous solution. The height of the water column in the incubation dish was kept at a value of 2 mm. The maximum average body height at the level of the swim bladder of the 5 dpf eleutheroembryos was 0.622 ± 0.042 mm (±SD, *n* = 13). The experimental system thus created the equivalent of a swimming tunnel, in which the animal can swim freely in the X-Y plane while minimizing the possibility of z axis deviation (see Video S4 as an example). For an EFP-induced escape response, a 20 V EFP was applied for 10 ms driving an ultrarapid motor response. EFP stimulation induced a well stereotyped response without loss of balance. Each EFP-induced escape motion was recorded with a Photron FASTCAM Mini WX100 high-speed camera through a Sigma 105 mm F2.8 EX DG lens. Videos were acquired at a frame rate of 10,000 frames per second with a spatial resolution of 1,000 dpi (512 × 420 pixels, covering a window of 13.0 × 10.7 mm^2^). The plate was illuminated from below by an LLUB White LED Blacklight 50 × 50 (PHLOX, Aix-en-Provence, France) adjusted to 5% using a Gardasoft RT 220-20 led light controller (Gardasoft, Cambridge, UK). The light intensity in the testing platform, measured using an ILT1400 radiometer (International Light technologies Inc., Peabody, MA, USA), was approximately 300 μW/cm^2^.

#### Kinematic analysis of experimental videos

The kinematics of the C-start escape response were similar when using tactile or EFP stimulation (Figures 2A, Video S1, Video S2 top panels; Figure S3, and Video S4). This indicates that no alteration in the neuromuscular system was induced under the conditions used for electrical stimulation. Viscous swimming media were prepared by dissolving Dextran 500 (Pharmacosmos A/S, Holbaek, Denmark) in filtered fish water resulting in an enhanced swimming fluid viscosity from 0.83 mPa⋅s (no dextran) to 15 mPa⋅s (9% dextran m/m) at 28°C without affecting other physical properties such as density.^13^^,^^14^^,^^69^^,^^70^ For each viscosity condition, 10 to 15 EFP-induced escape responses were recorded and a kinematic analysis was performed including quantification of mass center distance traveled, speed, rotation and angular velocity over six tail bend/beats using custom MATLAB or Python scripts. From the 10 to 15 individual acquired swims, three stereotyped escape responses were selected for further 3D processing. The primary criteria for choosing these escape responses were adequate imaging quality, a minimum number of tail bends/beats, no initial motion, a perfectly planar escape response with no roll or pitch motion, and the absence of contact between the tail and head during the swim.

#### Procrustes analysis

The first step in generating 3D energetic simulations was to apply a Procrustes analysis algorithm that subtracts translational and rotational motions from segmented zebrafish shapes across the experimental images over time. For each image, the fish shape was segmented through a simple thresholding under the lower percentile of pixel intensity. The segmented fish shape two-dimensional center of mass was then determined and used to center each frame. A custom algorithm based on the momentum equation was then used to derive the rotation (*α*) of the centered fish shape, similar to what has previously been described.^51^^,^^52^ After rotation of the first fish shape, the differential angle between consecutive images was computed in order to diminish rotation-induced discretization errors: zebrafish silhouette at time *t* is rotated by *α*_*t−*1_ before computation of the new body angle to vertical; this is performed iteratively until the escape response is completed. Mass center coordinates and body rotations are temporally smoothed by various MATLAB functions before centering on mass center and subtracting rotational displacements. Hence, the resulting snapshots exclusively describe the isolated body movements of the observed 2D zebrafish shape. It should be noted that the tracking method was mainly limited to 2D swimming cases, despite the fact that fish larvae tend to roll and pitch in addition to their yawing movements, especially during the short duration of the C-bend of fast-starts.^52^ However, the plane of body movement was considered to be approximately aligned with the X-Y plane for the total duration of the six-tail bends/beats measured during the experimental escape responses. This is made possible, on the one hand by the fact that the height of the water column in the incubation chamber was minimal compared to the height of the body (see above) and, on the other hand by the fact that any deviation along the z axis would be negligible compared to the total distance covered during the swimming cycles observed.

#### Midline tracking and body curvature assessment

From the zebrafish 2D isolated body movements, the anteroposterior axis was determined using custom skeletonization algorithms based on level-set gradient computation and head tracking. This midline was discretized into 300 segments. The angle between each consecutive segment was computed and averaged over all midline segments to obtain the midline curvature, an indicator of body movement amplitude.^51^^,^^52^ During escape responses, we observed high bending amplitudes during C-bends and counterbends followed by smaller periodic bending during the fast-swimming stage.

#### 3D reconstruction of zebrafish eleutheroembryo

For the present work, an approach was developed to reconstruct the zebrafish morphology in 3D, based on high-resolution histological cross-sections of 5 dpf zebrafish, generated by Hildebrand and collaborators.^47^ This database is composed of 18,627 transverse sections of zebrafish from the snout to the pectoral fins, hence describing the complex zebrafish shape. Since multiple cross-sections were missing, we used 203 high-resolution images, uniformly spaced by 4.8 μm to obtain a 3D zebrafish silhouette. All transverse slices were post-processed and binarized by automatically delimiting the body contour, imposing bilateral symmetry. At such early developmental stages, the anterior part of the body is much more complex and characteristic while the most posterior part can be reconstructed from transverse slices located at key positions regarding the specific geometry of the median fin-fold surrounding the tail. The final geometry of this median fold of integument, which extends along the eleutheroembryo body above and below it, was adjusted by comparison with images captured under the microscope (Figures 1C and 1D). In total, we constructed up to 34 key slices to obtain a standard body length, i.e., the distance between the tip of the snout and the end of the vertebral column, of 3.829 mm. Afterward, we performed multiple Wasserstein interpolations based on the Sinkhorn algorithm,^71^ to interpolate 1,602 transverse slices uniformly spaced at 2.4 μm along the length of the zebrafish. We found the computation of barycenters was well suited for shape interpolation with respect to a regularized Wasserstein metric. As a result, all sections have been normalized and thresholded to form a well-rounded 3D zebrafish volume. In addition, the pectoral fins were modeled as completely adducted to the body in accordance with the fast-start literature.^38^^,^^52^ This was confirmed by our own observations (Figure S3, and Video S4). During touch-induced fast-start escape responses, it is possible to anticipate the direction of escape, i.e., in the direction opposite to the touch. It was not possible to predict with certainty the direction of the motor response induced by an EFP, although the kinematics obtained were very similar to those of touch-induced escape responses (Figures 2A and S3). The touch response enables recordings of the pectoral fin movements at high resolution during the different phases of the C-start escape response. The animal alternates the beats of its pectoral fins to maintain its balance in the water column prior to stimulation (Figure S3, images 1 to 4, and Video S4) and fully adducts both of them from the start of the C-bend until the end of the fast-swimming stage (Figure S3, images 5 to 11, and Video S4). Therefore, the pectoral fins were modeled as fully adducted along the body during the EFP escape response.

As we used histological sections to reconstruct the body shape, we determined whether shrinkage might have occurred during histological treatments. The total body length of a 5 dpf zebrafish, i.e., the distance between the tip of the snout and the end of the caudal fin, was 4.076 ± 0.169 mm in live eleutheroembryos versus 3.954 ± 0.110 after fixation in 4% paraformaldehyde. In living 5 dpf zebrafish, the standard body length was 3.829 ± 0.158 mm and the length of the of the caudal fin portion of the median fin-fold was 0.247 ± 0.019 mm versus 3.707 ± 0.112 mm and 0.248 ± 0.020 mm, respectively, after fixation (± of SD, *n* = 11 to 13). Based upon the ratio of height to standard length, we scaled the 3D volume of a 5 dpf zebrafish at rest using a standard body length of 3.829 mm and formed by voxels sized 2.2517^2^ × 2.4 μm^3^ (Figure 1). We computed the level-set function associated with this 3D volume to generate a surface mesh that was bent to reproduce experimental planar movements originating from Procrustes analysis.

#### Reconstitution of escape body movements in 3D

The initial 3D volume was deformed to fit experimental movements of the midline originating from two-dimensional Procrustes analysis. To that end, a custom algorithm was implemented in order to track the anteroposterior extremities of the midline over time, by a computation of the level-set function and its gradient for each 2D segmented deformation. The 3D movement snapshots corresponding to each time step were reconstructed by deforming the midline of the initial volume and each transverse slice accordingly. In the present work, we assumed the Euler-Bernoulli principle: each transverse slice remains orthogonal to the midline across time. Thus, the initial surface mesh was generated to uniformly mesh the whole surface while associating each Lagrangian marker to a transverse slice across the midline. In addition, the experimental 2D silhouettes were used to rescale the standard length of the initial zebrafish 3D volume according to the size of each zebrafish observed experimentally. Our methodology demanded three strict conditions regarding the experimental imaging. First, we assumed that the zebrafish and the surrounding flow are initially in a resting state. Secondly, we assumed that movements were planar, i.e., pitch and roll motions were considered negligible, in order to reconstruct the 3D movement snapshots via the Euler-Bernoulli theory, as images were recorded from one camera located above the fish. Thirdly, the time and spatial resolutions have to be high enough to precisely track the zebrafish midline over time. As a consequence, we used a high-resolution ultra-fast camera and we paid specific attention to both the initial state before the stimulation and during the subsequent movements. After reconstructing the moving 3D zebrafish shape, the 3D zebrafish shapes were centered and rotated at each time step by performing a second Procrustes analysis based on 3D mass center and rotation. Finally, the 3D movement snapshots were temporally smoothed using a mean filter over a window of 0.5 ms on the position of every Lagrangian marker that described the 3D surface.

#### Energetic simulations by resolution of Navier-Stokes equations

The 3D movement snapshots reproducing escape responses were entered in the Navier-Stokes solver by immersion in a fluid of chosen viscosity. We considered that body movements could be estimated directly from experimental imaging. The images were recentered and rotated so that momentum and angular momentum were constant. Consequently, only the body movement kinematics of the fish larvae were recovered from the images, and not the fish displacement dynamics. Therefore, no 3D shape properties such as elastic feedback were implemented to replicate the observed body movements. We used an in-house Navier-Stokes implementation for solving the fluid-structure interaction problem. Basically, we used a numerical method based on a Cartesian mesh, able to handle complex solid geometry and deformations, by solving the penalized Navier-Stokes equations in the whole computational domain, i.e., both fluid and solid regions:

$$\frac{\partial u}{\partial t}+\left(u\;\cdot \nabla \right)u=-\frac{1}{\rho }\nabla p+\frac{1}{\rho }\nabla \cdot \;2\mu D\left(u\right)+\chi_{s}\lambda \left(u_{s}-u\right)$$

∇·u=0

where u and $u_{s}$ are the tri-dimensional velocity of the fluid and solid regions, respectively, $D=\frac{\nabla u+\nabla^{T}\;u}{2}$ is the diffusion tensor, *ρ* and *μ* are the density and dynamic viscosity of the fluid, *p* is the pressure, $\chi_{s}$ is the dimensionless characteristic function of the solid, and *λ* is its penalty coefficient with dimensions of s^−1^. For more details about our Navier-Stokes solver, please refer to the following referenced reports.^26^^,^^29^^,^^46^^,^^50^

The numerical simulation was performed in the international system dimensional units, i.e., viscosity was given in $m^{2}/s$, lengths in meters and densities in $kg/m^{3}$, etc. Numerical schemes aimed to ensure that the approximated solution converged to the exact solution as the grid resolution (Δx→0) increased. However, a balance had to be found between numerical accuracy and mesh size to manage computational cost effectively. The grid convergence study was conducted in 2D to keep the computational cost affordable. A grid size of $\Delta x=2.857\times 10^{-2}\;mm$ (Nx=420) provided a reasonable trade-off. Higher resolutions (Nx=525) have been tested but they result in only marginal improvements in energetic accuracy, while coarser grids (Nx=315) show slight deviations, particularly during the high-speed swimming phase.^72^

In the 3D simulations, a uniform Cartesian mesh with a grid size of $\Delta x=2.857\times 10^{-2}\;mm$ in all three directions was therefore used. The simulation domain measured 12×12×2mm, with the center of the domain aligned with the center of mass of the larvae at the initial time. The fin folds were represented with at least two grid points in the thickness direction.

#### Sampling and quantification of kinematic and energetic parameters during simulations

Unlike previous numerical methods,^26^^,^^29^^,^^46^^,^^50^ the present work was focused on describing the true shapes $\chi_{s}$ and body movements $\tilde{u}$ that drive real zebrafish escape responses. To bypass mathematical modeling and accurately model the motion of the zebrafish, Lagrangian markers associated with the 3D snapshots of body bending directly from the experimental observations were used as input to the Navier-Stokes solver (see ref. 50 for more details). The Lagrangian markers were computed to discretize and integrate surface elements across the solid body. These markers then helped compute surface velocity of the body $u_{s}$, which in the case of a deforming solid, combines rigid body kinematics with deformation velocity. Consistent with the publications of Bergmann and collaborators,^26^^,^^29^^,^^46^^,^^50^ the body surface velocity was decomposed as:

$$u_{s}\left(x,t\right)=\bar{u}\left(x,t\right)+u^{\theta }\left(x,t\right)+\tilde{u}\left(x,t\right)$$

where $\bar{u}$ was the translation velocity of the center of mass, $u^{\theta }$ was the rotation velocity around the center of mass given by $u^{\theta }=\omega \wedge r$, where r was the position vector of a surface element relative to the center of mass. The translation velocity $\bar{u}$ of the center of mass and the angular velocity ω around the center of mass were governed by the equations of motion for translation and rotation:

$$\rho_{s}V_{s}\frac{d\bar{u}}{dt}=F^{\text{hydro}}$$

$$\frac{dJ\omega }{dt}=M^{\text{hydro}}$$

where $\rho_{s}$ was the solid mass density (which was approximated to ρ), $V_{s}$ the solid volume, J the inertia matrix and $F^{\text{hydro}},M^{\text{hydro}}$ represented the hydrodynamic forces and torques exerted upon the solid body.

Hydrodynamic forces and torques were calculated by integrating the hydrodynamic tensor T over the body surface elements. The stress tensor depended on the fluid’s pressure p, the velocity field u**,** and fluid dynamic viscosity μ (which was constant during the simulation):

$$T\left(u,p\right)=-pI+\mu \left(\nabla u+\nabla u^{T}\right)$$

The hydrodynamic force $F^{\text{hydro}}$ and torque $M^{\text{hydro}}$ were then given by:

$$F^{\text{hydro}}=-\oint_{\Gamma_{s}}T\left(u,p\right)\cdot n\text{dS}$$

$$M^{\text{hydro}}=-\oint_{\Gamma_{s}}T\left(u,p\right)\wedge r\text{dS}$$

The instantaneous power P exerted by the zebrafish was derived from the computed body boundary velocity $u_{s}$ and the hydrodynamic tensor T:

$$P=\int_{\Gamma_{s}}\left(-\oint_{\text{dS}}T\left(u,p\right)\cdot n\delta S\right)\cdot u_{s}\text{dS}$$

where **n** was the outward unit normal vector to the body surface. The inclusion of $\bar{u}$, $u^{\theta }$ and $\tilde{u}$ in $u_{s}$ implicitly incorporated the effects of translation, torque and body movement on power consumption. This power therefore accounted for both passive (body displacement) and active (body deformation) contributions to movement. Assuming no external energy sources influenced the fluid flow or the fish’s movements, the quantified power represented the mechanical power exerted by the zebrafish body to overcome surrounding forces and propel itself through the media. Although this power measurement represented the mechanical energy expended for movement over time, it did not capture the entire scope of chemical energy consumption in muscle tissues, including the conversion of chemical energy into heat.

To determine the total work $W_{total}$, corresponding to the total energy expended by the zebrafish over a time interval from $t_{i}$ to $t_{f}$, the instantaneous power was integrated over time:

$$E=W_{total}=\int_{t=t_{i}}^{t_{f}}Pdt\approx \sum_{t=t_{i}}^{t_{f}}P_{i}dt$$

where $P_{i}$ represented the power at discrete time steps, and dt the time increment.

The total amount of work is often employed to analyze the energetic efficiency of a system by separating the useful work from the rest, for example through the computation of Froude efficiency defined as:

$$\eta_{Fr}=\frac{W_{useful}}{W_{total}}$$

While simple deformation laws, such as harmonic traveling waves, allow for straightforward computation of useful work, complex body movements like those seen in zebrafish escape responses make it challenging to isolate the most significant contributions to propulsion. Consequently, an alternative performance metric designated cost of transport (CoT), defined as the ratio of total energy over the total distance traveled was computed:

$$\mathrm{CoT}=\frac{E}{d_{total}}$$

where $d_{total}$ is the total distance traveled between time $t_{i}$ and time $t_{f}$ in the sense of the cumulative sum of distance traveled across time. The CoT is particularly attractive for escape kinematics as it measures the energetics invested by zebrafish per distance moved by its center of mass.

During the numerical simulations, each quantified kinematic or energetic parameter was computed and saved at each time step. At 10,000 frames per second, 500 to 800 total time periods were required to simulate the six-tail bends/beats as their total duration was between 50 and 80 ms. As a result, some computed dynamic quantities such as trajectory, velocity or power were compromised by signal noise. To reduce such noise, kinematic and energetic data were smoothed through post-processing steps following the numerical simulation including a combination of a Savitzky-Golay filter and spline interpolations (from the *scipy* python module).^72^ The mechanical power expended at each experimental time step was averaged over a window of one millisecond, and can be visualized across all time steps (e.g., Figures 5C and 5F, red line). From the power output, we computed the mean power value by averaging the smoothed power over time during the six tail-movement cycles of fast-starts and the total expended energy during these six movements by integration of the area of the smoothed mechanical power curve (as explained above).

#### Reynolds number

The dimensionless Reynolds number (Re) can be used to analyze the relative influence of viscous and inertial forces during motion in a fluid. Re serves as an indicator of the flow regime, ranging from laminar and viscous-dominated flows to turbulent and inertial-dominated flows. At low Re, viscous forces predominate resulting in smooth, orderly flow. Conversely, at high Re, inertial forces become significant, often leading to nonlinear effects and turbulence.

In this study, Re was calculated based on the simulation results using the formula:

$$Re=\frac{\rho V_{\text{mean}}L}{\mu }$$

where ρ was the fluid density, $V_{\text{mean}}$ the mean computed speed during the six simulated tail beats of the escape response, L the total length of the simulated zebrafish measured in the experimental images, and μ the dynamic viscosity of the fluid.

#### 3D visualization of wake: Q-criterion

Flow vorticity was defined as the quantitative variable which measured the rotation of the flow to enlighten where the vortices were located in the wake. Thus, the fluid vorticity was given by:

Ω=∇∧u

For 3D visualization of vortices generated in the wake during each fast-start, we computed the Q-criterion defined as:^73^

$$Q=\frac{1}{2}\left(\;\|\Omega {\;\|}^{2}-\;\|\Phi {\;\|}^{2}\right)$$

where Ω, and Φ were the asymmetric and symmetric components of the velocity gradient, respectively, which also represented the vorticity and rate of strain tensors. The Q-criterion is often preferred to the vorticity when illustrating the generated wake behind a self-propelled swimmer.

### Quantification and statistical analysis

#### Statistical analysis

Statistical analyses were performed using GraphPad Prism 8 Software (GraphPad, Boston, US). Gaussian distribution of compared groups was tested with the Shapiro-Wilk test, and homogeneity of variances between groups were assessed via Levene’s test. All tested groups of values did not significantly differ from normality and had non-significantly different variances (*p* > 0.05). Differences between groups was tested using a two-way ANOVA test with Tukey’s multiple comparisons or compared to controls using Dunnet’s post-hoc test. A two-way ANOVA test with Sidak’s multiple comparisons was used to determine the significance between measured kinematic parameters of original escape response movies and numerical simulations. The statistical difference in linear slopes was also assessed. For all of the statistical results, the significance thresholds were ∗*p* < 0.05, ∗∗*p* < 0.01, ∗∗∗*p* < 0.001, and ∗∗∗∗*p* < 0.0001.
