# Supplementary material for: Modeling zebrafish escape swim reveals maximum neuromuscular power output and efficient body movement adaptation to increased water viscosity
Source: iScience. 2025 Feb 17;28(3):112056. doi: 10.1016/j.isci.2025.112056 (PMC11930232; doi:10.1016/j.isci.2025.112056)
Supplement: Document S1. Figures S1–S3 [file mmc1.pdf]

iScience, Volume *28*

Supplemental information

Modeling zebrafish escape swim reveals maximum neuromuscular power output and efficient body movement adaptation to increased water viscosity

Guillaume Ravel, Théo Mercé, Michel Bergmann, Anja Knoll-Gellida, Afaf Bouharguane, Sara Al Kassir, Angelo Iollo, and Patrick J. Babin

SUPPLEMENTAL INFORMATION

SUPPLEMENTAL FIGURES

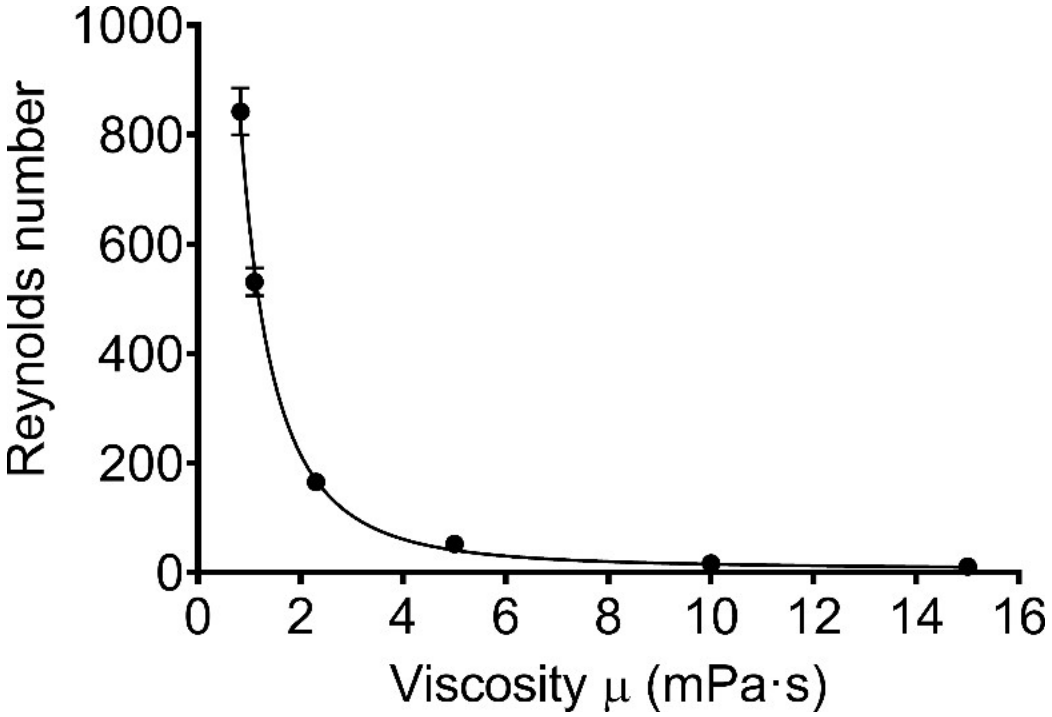

Figure S1. **Reynolds number of 5 dpf zebrafish fast-starts as a function of fluid viscosity.** Data represents mean ± SEM of three escape responses per viscosity condition. The Reynolds number computation was based on the mean velocity estimated by the experiment-driven numerical simulations.

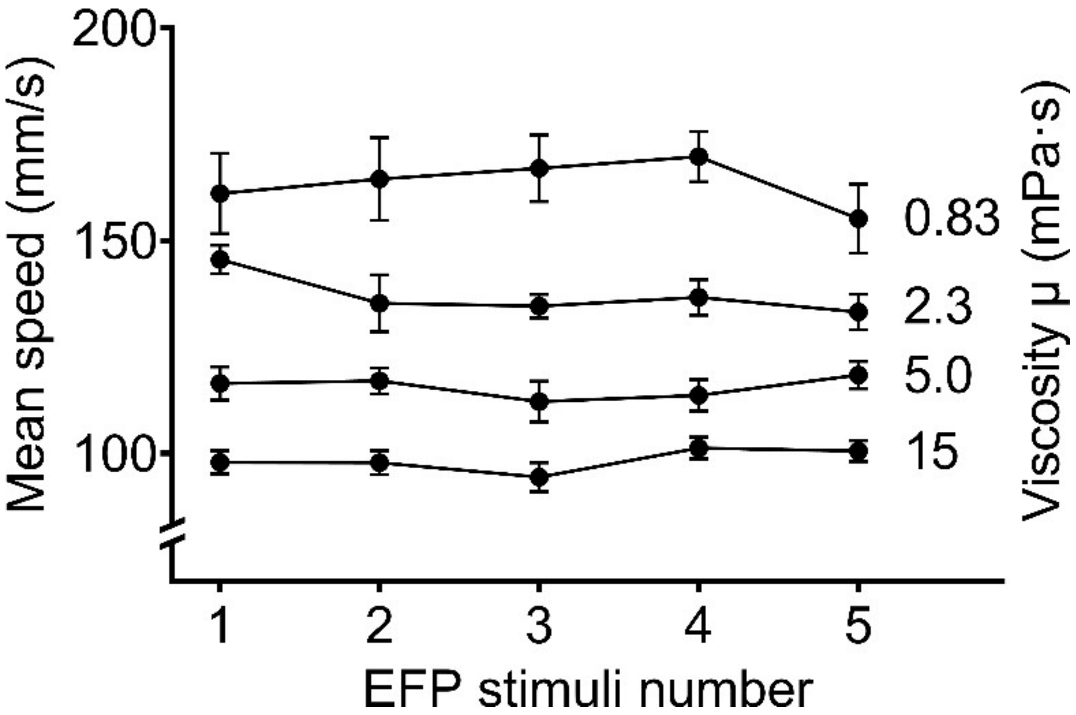

Figure S2. **Eleutheroembryos did not display habituation or fatigue after several consecutive EFP-induced fast starts.** Five consecutive EFP-induced escape responses of 5 dpf eleutheroembryos in groups of 10 to 12 individuals were recorded by a high-speed Photron Fastcam SA3 camera (Photron USA Inc., San Diego, CA, USA) at a resolution of 512 x 512 pixels, at 1,000 frames per second while being illuminated from below. Delay between EFP-stimuli was approximately 15 seconds. Traveled distance for each fish during 100 ms was extracted automatically using a homemade tracking software; and converted to average speed. Fish that could not be tracked for 100 ms or that did not react within a time-lapse of 20 ms after the EFP stimuli were excluded. For each viscosity, the displayed data corresponds to mean ± SEM of mean speed by six groups of eleutheroembryos from three independent breedings, accounting for 22 to 39 fast starts per EFP stimuli. Linear regression analysis showed that slopes for all four viscosity data sets were not significantly different from zero, hence supporting the conclusion that five consecutive EFP-induced fast starts resulted in neither habituation nor fatigue.

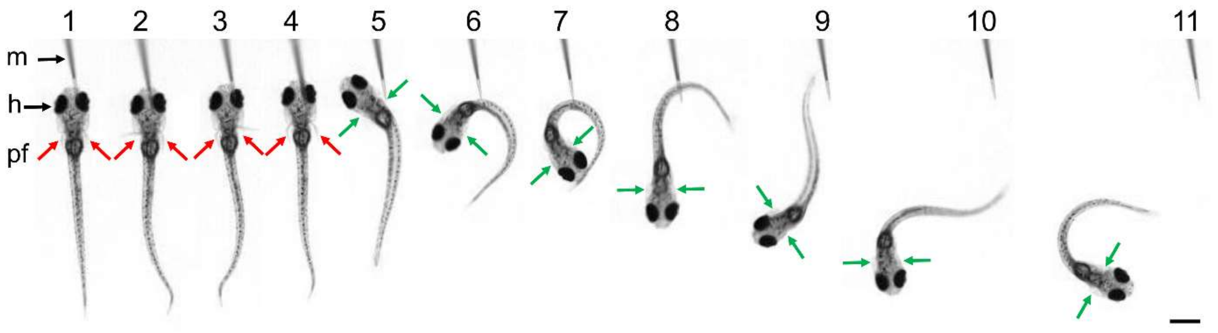

Figure S3. Position of pectoral fins during a touch-induced C-start escape response of a pre-feeding zebrafish larva. A tactile stimulation was applied to the upper aspect of the head to induce the fast-start escape swim. The animal alternated the beats of its pectoral fins (pf) to maintain its balance in the water column prior to stimulation (images 1 to 4, red arrows) and completely adducted them as soon as the escape swim began (images 5 to 11, green arrows). The images shown were extracted from a film acquired at 2,000 frames/second (Video S4). Other abbreviations: h, head; m, micropipette. Scale bar: 500 µm.
